# Supplementary material for: Proximity proteomics reveals a co-evolved LRRK2-regulatory network linked to centrosomes
Source: EMBO Rep. 2026 May 23;27(12):3488–512. doi: 10.1038/s44319-026-00806-4 (PMC13304329; doi:10.1038/s44319-026-00806-4)
Supplement: Supplementary file 1 — Appendix [file 44319_2026_806_MOESM1_ESM.pdf]

Appendix for “Proximity proteomics reveals a co-evolved LRRK2-regulatory network linked to centrosomes”

Table Of Contents

Appendix Figure S1: Western blot, pRab10, LRRK2-pS935 and biotinylation – page 2.

Appendix Figure S2: ROC Curve of prediction with Mutual Information (MI) of LRRK2’s IntAct interaction types – page 3.

Appendix Figure S3: Heatmap with cluster specific, significantly enriched processes obtained from co-evolution-based clustering of the LRRK2 BioID interactome – page 4.

Appendix Figure S4: Heatmap displaying the co-evolution-based hierarchical clustering of the LRRK2’s IntAct interactome – page 5.

Appendix Figure S5: Effect of CYLD co-expression and ubiquitin derivatives on steady-state LRRK2 levels – page 6.

Appendix Figure S6: Western blot analysis demonstrating effective LRRK2 inhibition by MLi-2/GZD-824; volcano plot/ proximity proteome MLi-2 vs GZD-824; validation of PCM1 knock-down by western blot – page 7.

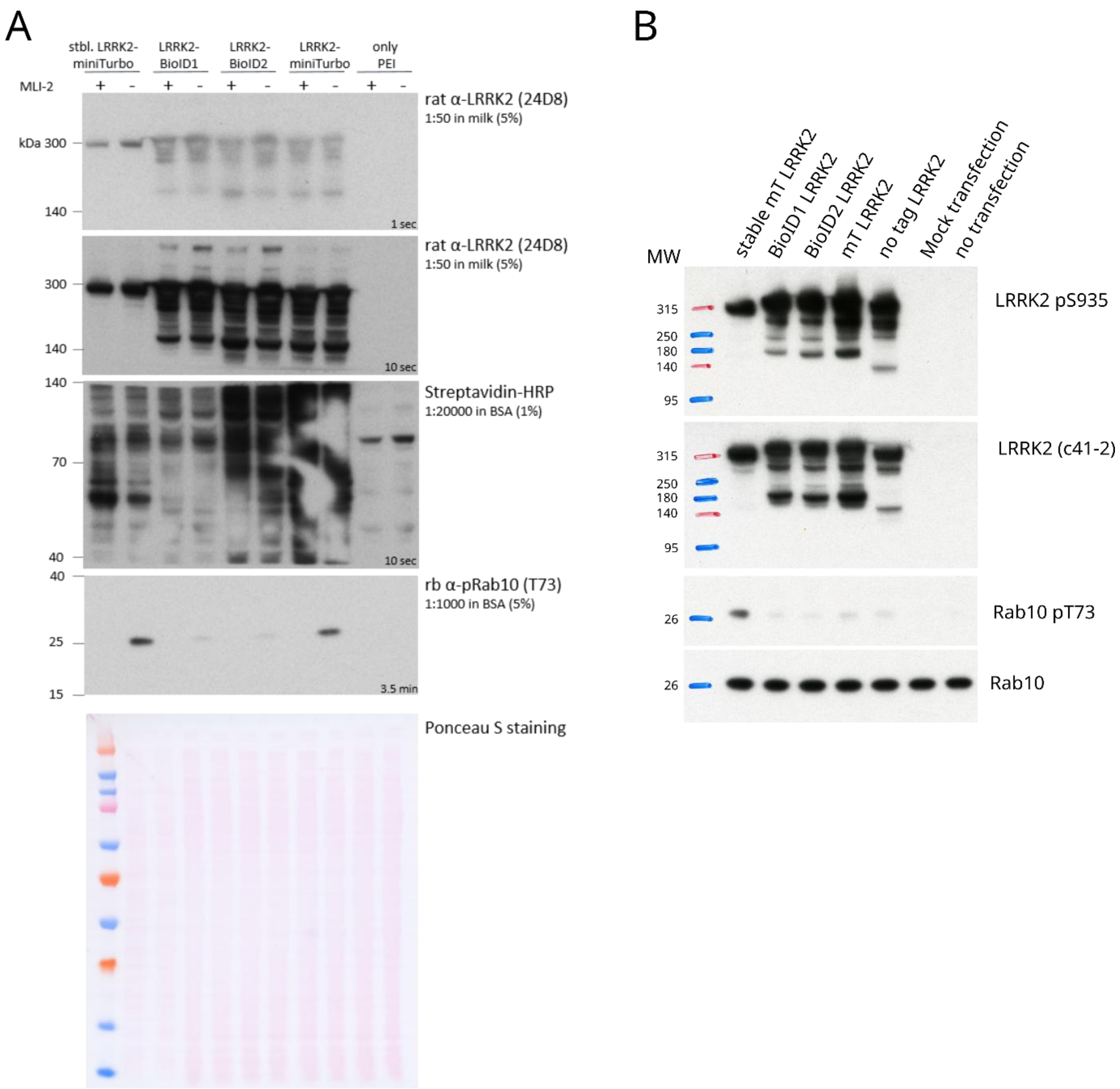

Appendix Figure S1: Western blot, pRab10, LRRK2-pS935 and biotinylation – comparison BioID1, BioID2, miniTurbo. To ensure functional BioID-fusion proteins, LRRK2 phosphorylation at S935 and Rab10 phosphorylation was assessed. (A) The different BioID LRRK2 constructs show sufficient biotinylation of proteins as well as MLi-2-responsive Rab10 phosphorylation. (B) All constructs are phosphorylated at S935 (top panel). Replication of LRRK2-mediated Rab10 phosphorylation (last panel) demonstrating functional BioID fusion proteins.

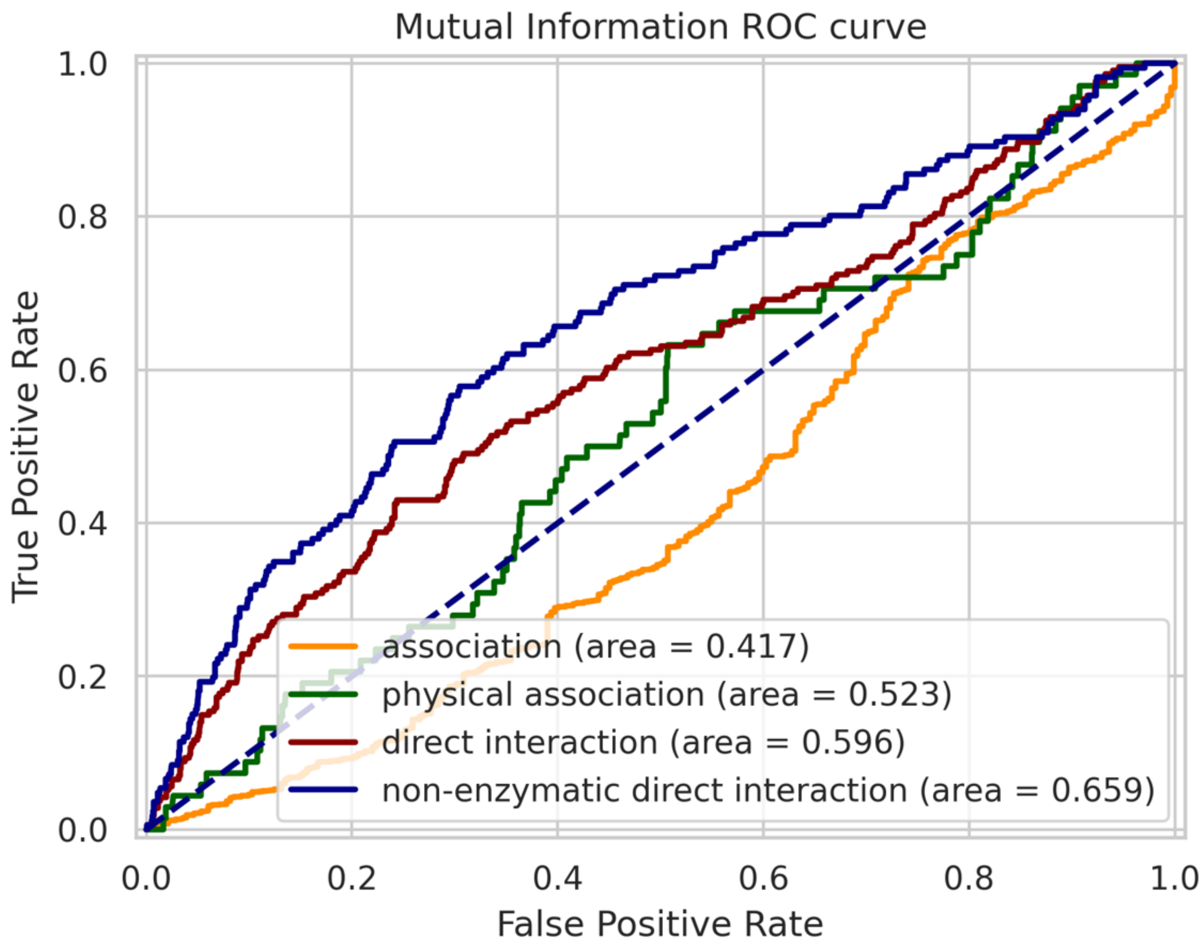

Appendix Figure S2: ROC Curve of prediction with Mutual Information (MI) of LRRK2's IntAct interaction types.

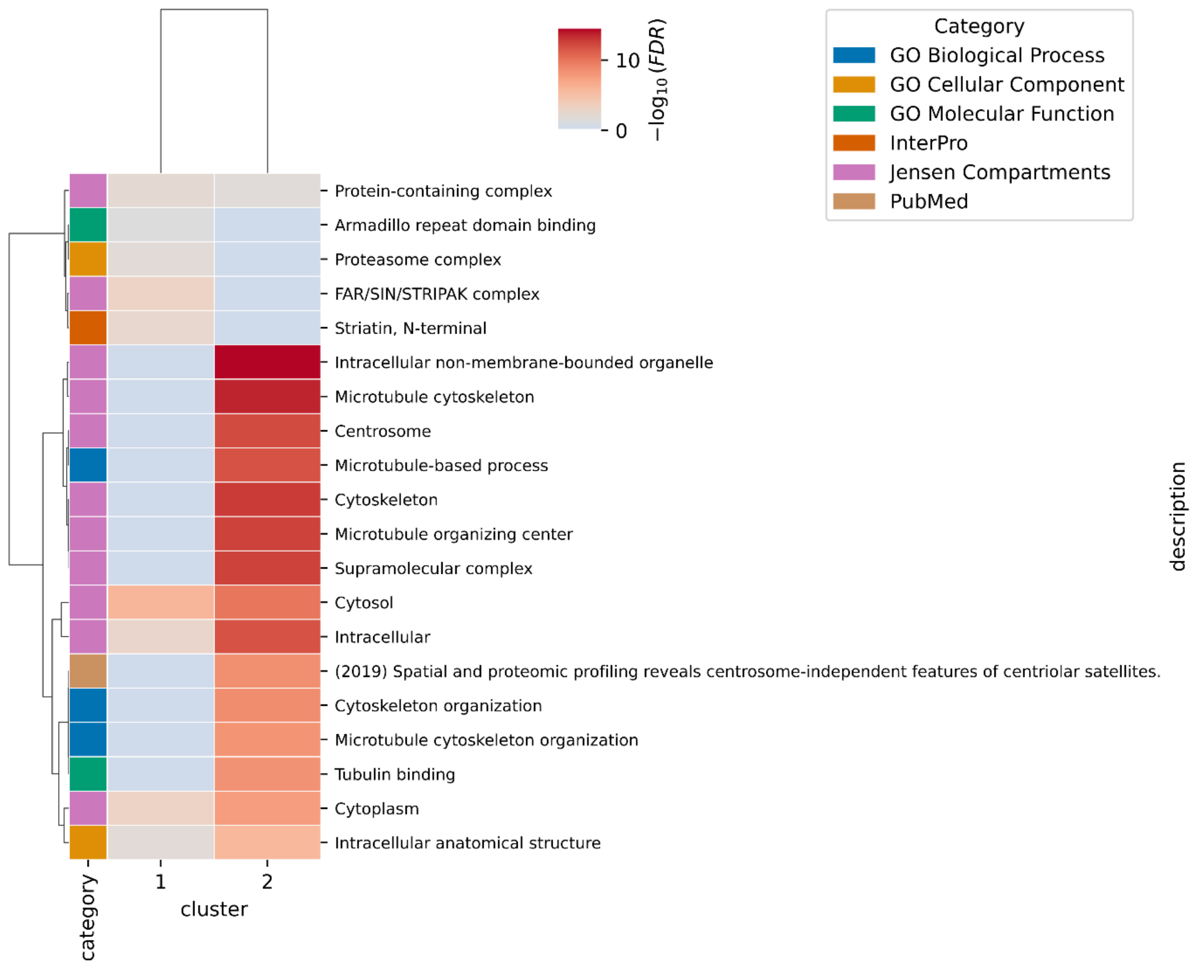

Appendix Figure S3: Heatmap with cluster specific, significantly enriched processes (FDR < 0.01; StringDB) obtained from co-evolution (Jaccard)-based clustering of the LRRK2 BioID interactome.

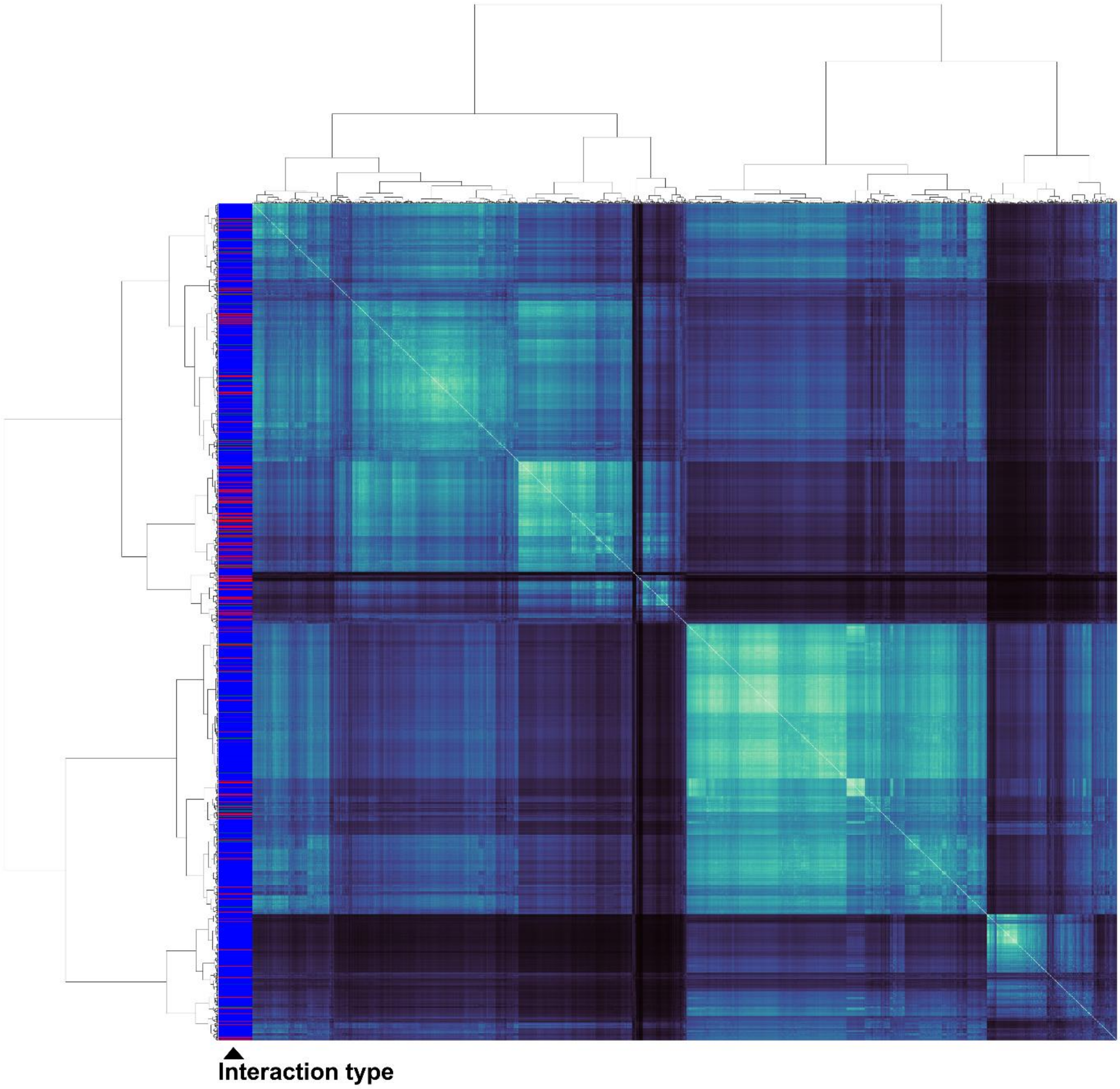

Appendix Figure S4: Heatmap displaying the co-evolution (Jaccard)-based hierarchical clustering of the LRRK2's IntAct interactome. Colored bars provide IntAct's interaction type annotation.

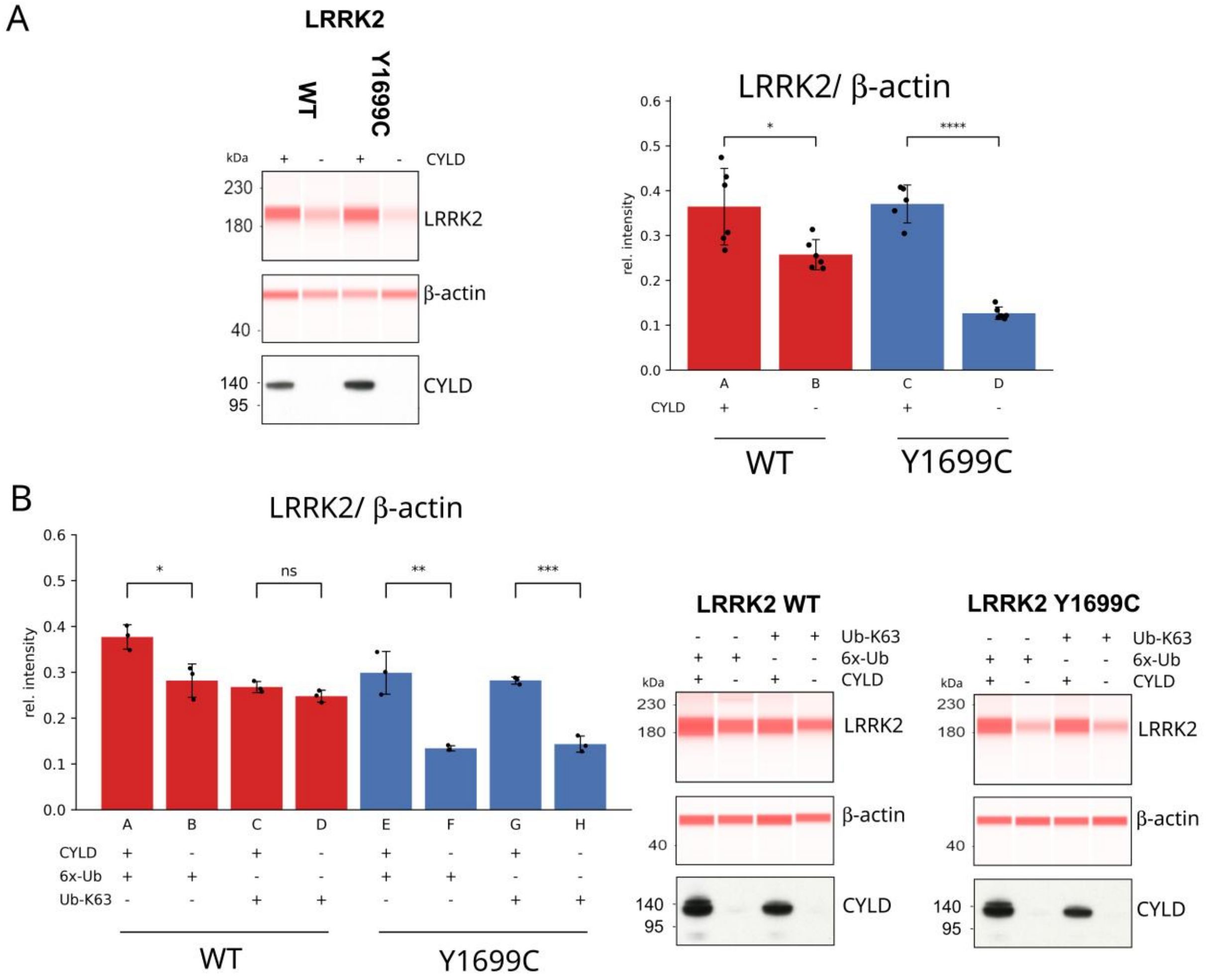

Appendix Figure S5: Effect of CYLD co-expression and ubiquitin derivatives on steady-state LRRK2 levels in WT and Y1699C variants (A) Effect of CYLD co-expression on steady-state LRRK2 levels (second independent experiment). LRRK2 WT or Y1699C was co-expressed with CYLD or empty vector control. LRRK2 protein levels were normalized by β-actin. Statistical significance was assessed using unpaired two-tailed t-tests (P-values: A-B: 0.0171; C-D: 3.0E-7; N = 6 biological replicates (for the Y1699C condition, one data point was skipped for technical reasons); error bars = SD) (left panel). Reconstructed Capillary Western signals and confirmation of CYLD expression by Western blot. On the capillary Western blot, LRRK2 migrates at a lower molecular weight (right panel). (B) Effect of ubiquitin conjugation type. LRRK2 WT or Y1699C was co-expressed with CYLD or empty vector control either in the presence of WT ubiquitin or K63-only ubiquitin (Lim *et al.*, 2005). Quantification of LRRK2 protein levels from three biological replicates. LRRK2 protein levels were normalized by β-actin. Statistical significance was assessed using unpaired two-tailed t-tests (P-values: A-B: 0.0218; C-D: 0.1289; E-F: 0.0037; G-H: 0.0002; N = 3 biological replicates; error bars = SD) (upper panel). Reconstructed Capillary Western signals and confirmation of CYLD expression by Western blot (lower panel).

A

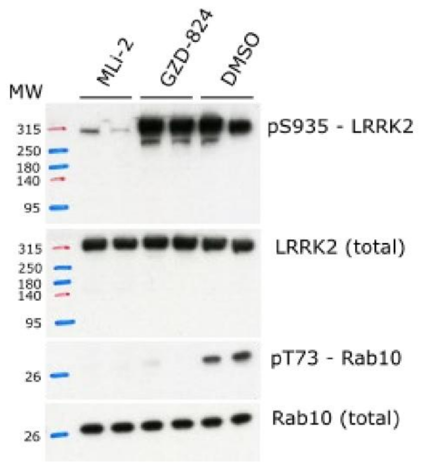

B

BioID: MLi-2 vs GZD824

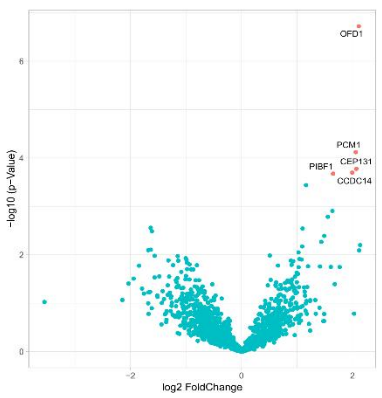

C

PCM1 knock-down/ Lysate controls

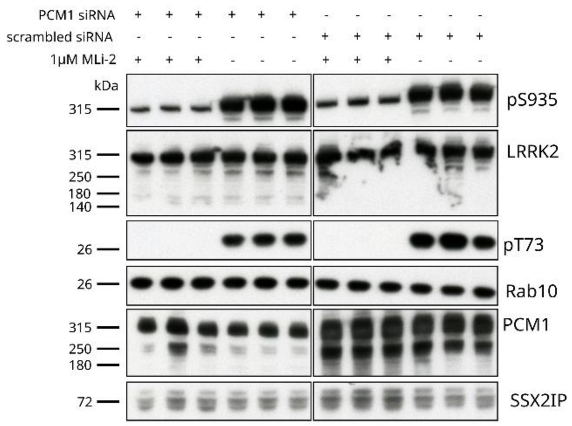

Appendix Figure S6: (A) Western blot analysis demonstrating effective LRRK2 inhibition by MLi-2 (type I inhibitor) and GZD-824 (type II inhibitor) in HEK293 cells stably expressing miniTurbo-tagged LRRK2. Both compounds at 1 µM significantly reduce Rab10 phosphorylation. As expected, MLi-2 induces a marked loss of LRRK2 pS935 phosphorylation, a hallmark of type I kinase inhibition. In contrast, GZD-824 does not affect pS935 phosphorylation, consistent with its classification as a type II inhibitor (Tasegian *et al.*, 2021). (B) BioID-based proximity labeling comparing the interactome changes upon treatment with MLi-2 versus GZD-824 (moderated permutation-based T-test, FDR = 0.05; S0 = 0.1; N = 7). Volcano plot illustrates differential protein interactions, highlighting the distinct molecular effects of type I and type II LRRK2 inhibition. (C) Validation of PCM1 knock-down by western blot. Efficient depletion of PCM1 is confirmed, with no observed changes in total protein levels of LRRK2, Rab10, or SSX2IP. LRRK2 inhibition by MLi-2 remains effective in the context of PCM1 knock-down.
