# Supplementary figures and images for: Proximity proteomics reveals a co-evolved LRRK2-regulatory network linked to centrosomes
Source: EMBO Rep. 2026 May 23;27(12):3488–512. doi: 10.1038/s44319-026-00806-4 (PMC13304329; doi:10.1038/s44319-026-00806-4)

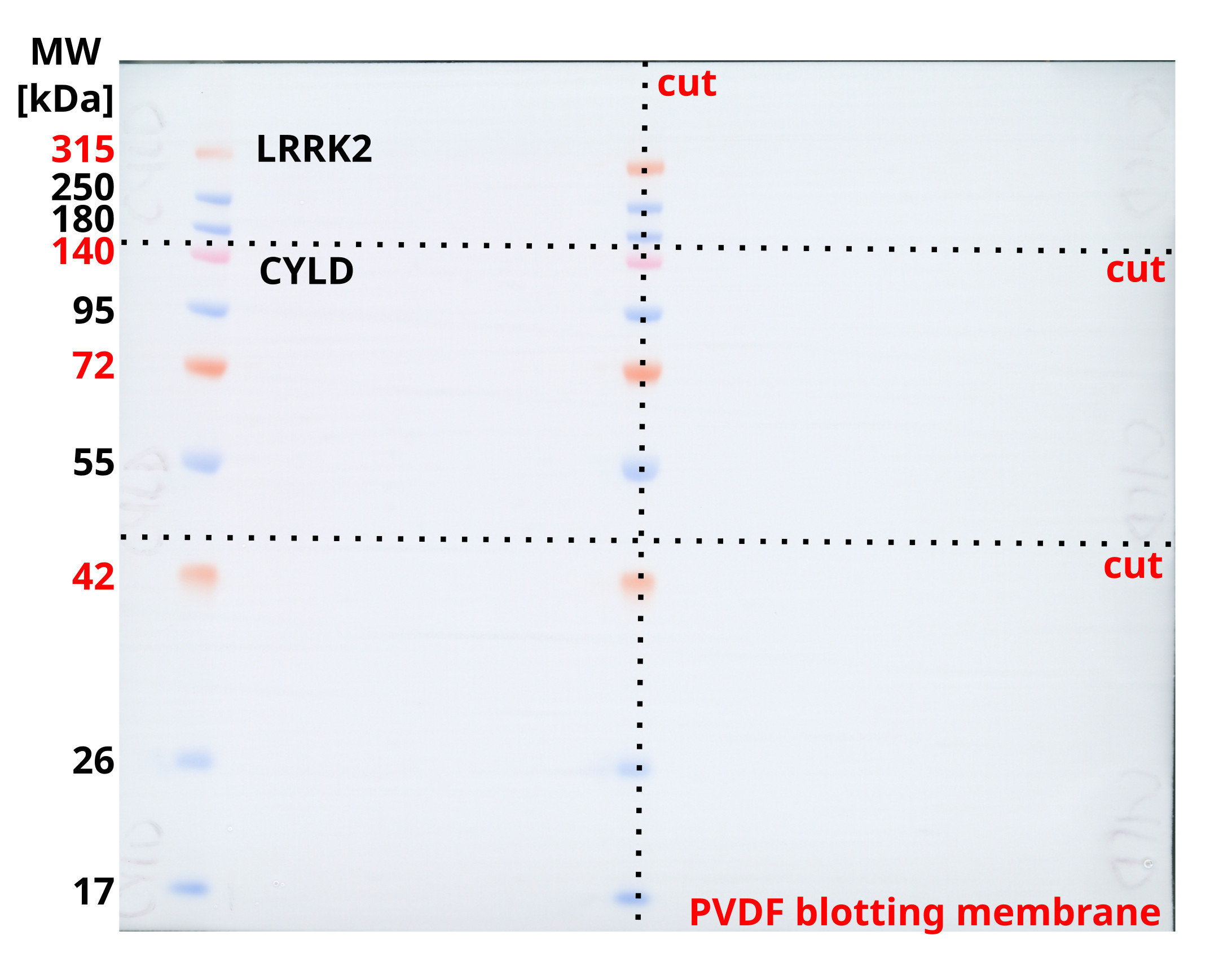

Supplement: Supplementary file 5 — Source data Fig. 3 [file 44319_2026_806_MOESM5_ESM.zip › Figure 3/3F/MW+slices.jpg]

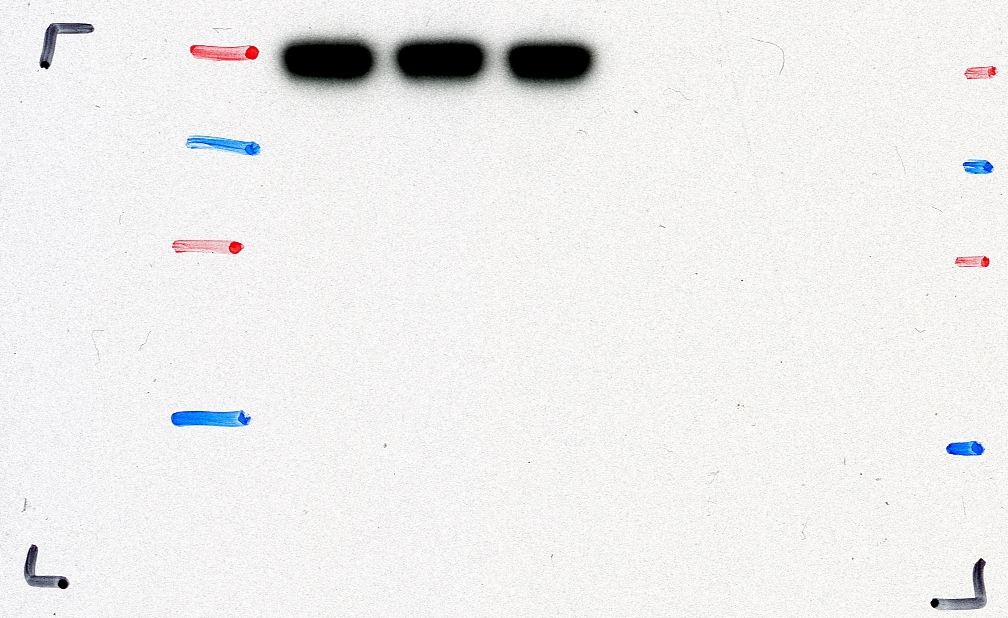

Supplement: Supplementary file 5 — Source data Fig. 3 [file 44319_2026_806_MOESM5_ESM.zip › Figure 3/3F/western CYLD.jpg]

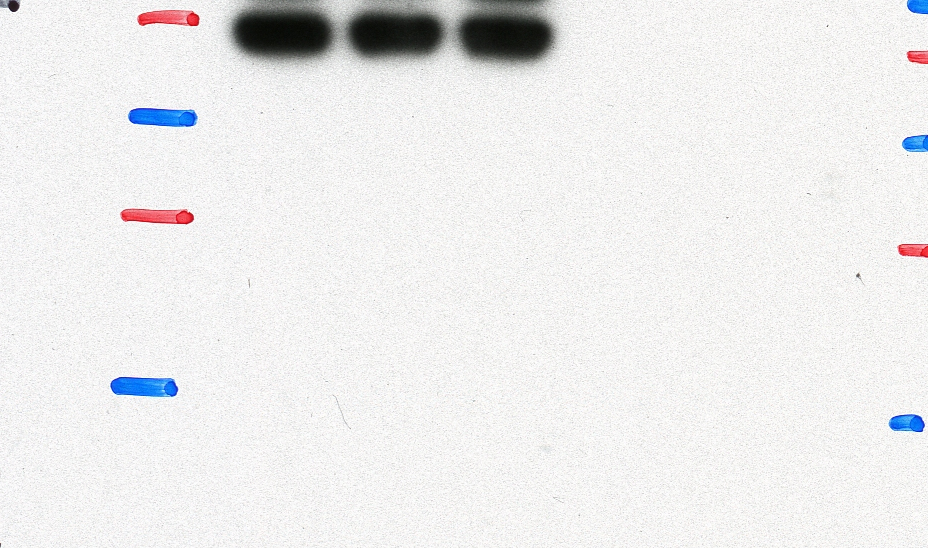

Supplement: Supplementary file 5 — Source data Fig. 3 [file 44319_2026_806_MOESM5_ESM.zip › Figure 3/3F/western FLAG.jpg]

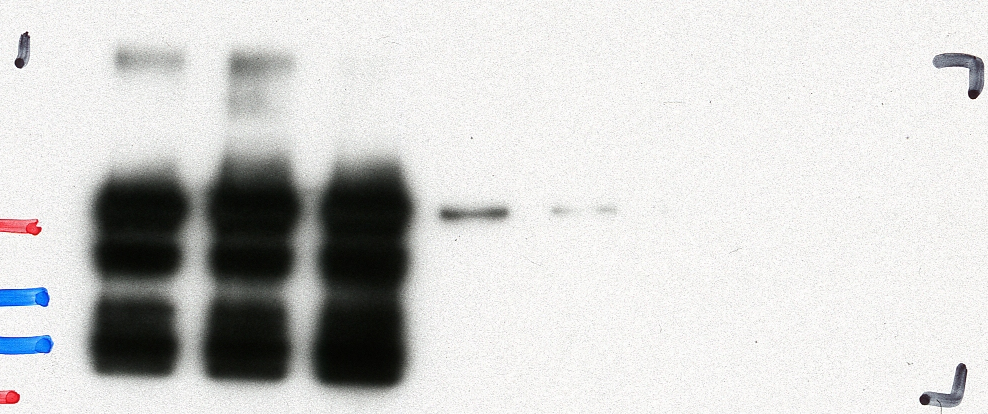

Supplement: Supplementary file 5 — Source data Fig. 3 [file 44319_2026_806_MOESM5_ESM.zip › Figure 3/3F/western LRRK2-24D8.jpg]
