# Supplementary material for: Proximity proteomics reveals a co-evolved LRRK2-regulatory network linked to centrosomes
Source: EMBO Rep. 2026 May 23;27(12):3488–512. doi: 10.1038/s44319-026-00806-4 (PMC13304329; doi:10.1038/s44319-026-00806-4)
Supplement: Supplementary file 6 — Expanded View Figures [file 44319_2026_806_MOESM6_ESM.pdf]

Expanded View Figures

Q17

Figure EV1. (**A**) interaction fingerprint clustering of LRRK2’s modulators (MLi-2 and RAB29) interactomes. Cells contain the minimum expected distance obtained from the distograms of any residue of each interactor (row) to every residue in LRRK2 (column). Rows are color annotated based on confidence score, Jaccard, RMSD from reference LRRK2 structure (PDB: 7LHW), and experiment type (either MLi-2 or RAB29 overexpression); (**B**) interaction fingerprint of LRRK2 and interacting RABs (from IntAct).

© The Author(s)

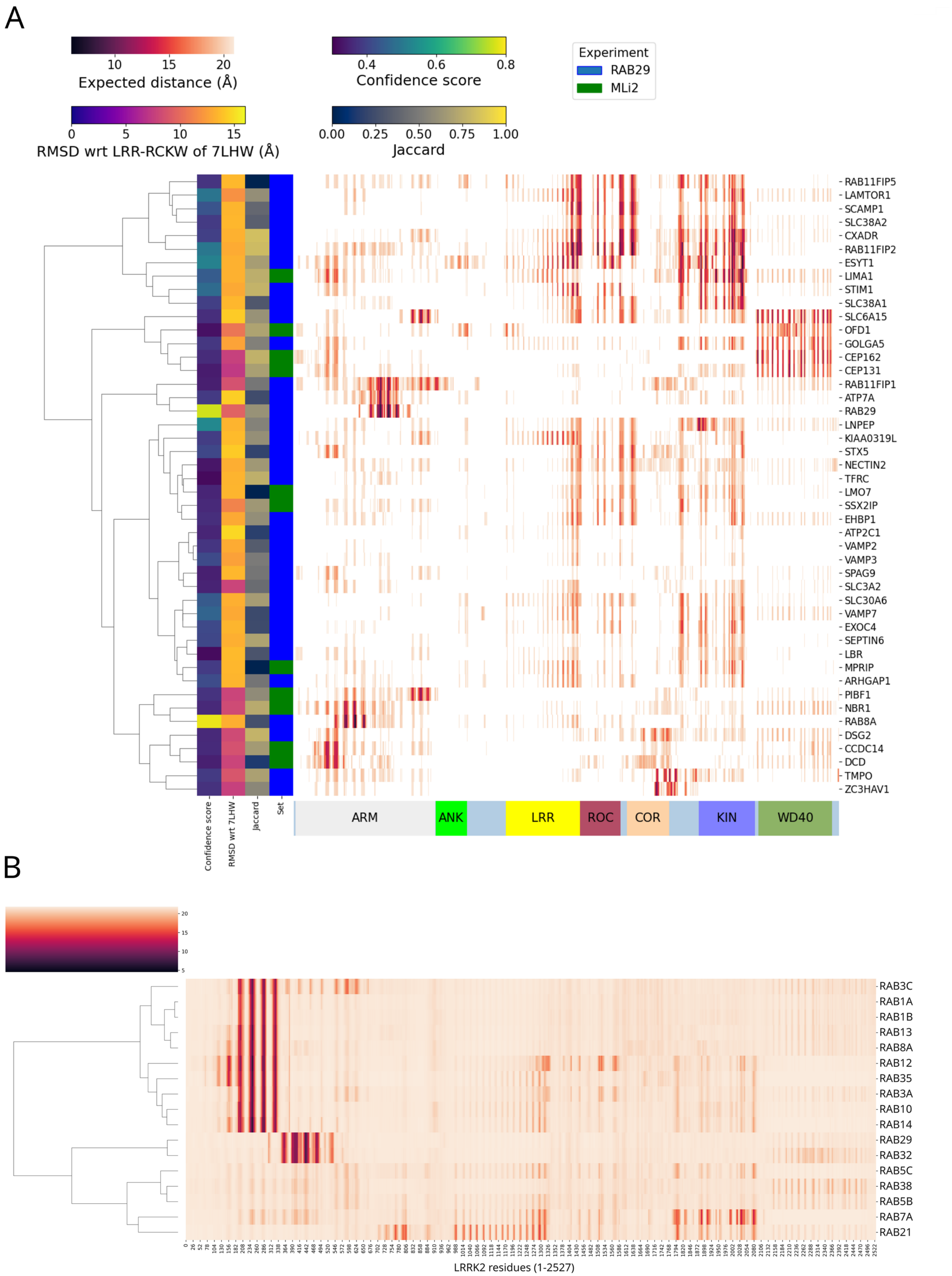
A
10 15 20
Expected distance (Å)
0.4 0.6 0.8
Confidence score
Experiment
RAB29
MLi2
0 5 10 15
RMSD wrt LRR-RCKW of 7LHW (Å)
0.00 0.25 0.50 0.75 1.00
Jaccard
RAB11FIP5
LAMTOR1
SCAMP1
SLC38A2
CXADR
RAB11FIP2
ESYT1
LIMA1
STIM1
SLC38A1
SLC6A15
OFD1
GOLGA5
CEP162
CEP131
RAB11FIP1
ATP7A
RAB29
LNPEP
KIAA0319L
STX5
NECTIN2
TFRC
LMO7
SSX2IP
EHBP1
ATP2C1
VAMP2
VAMP3
SPAG9
SLC3A2
SLC30A6
VAMP7
EXOC4
SEPTIN6
LBR
MPRIP
ARHGAP1
PIBF1
NBR1
RAB8A
DSG2
CCDC14
DCD
TMPO
ZC3HAV1
Confidence score
RMSD wrt 7LHW
Jaccard
Set
ARM
ANK
LRR
ROC
COR
KIN
WD40
B
RAB3C
RAB1A
RAB1B
RAB13
RAB8A
RAB12
RAB35
RAB3A
RAB10
RAB14
RAB29
RAB32
RAB5C
RAB38
RAB5B
RAB7A
RAB21
LRRK2 residues (1-2527)
