# Supplementary material for: Proximity proteomics reveals a co-evolved LRRK2-regulatory network linked to centrosomes
Source: EMBO Rep. 2026 May 23;27(12):3488–512. doi: 10.1038/s44319-026-00806-4 (PMC13304329; doi:10.1038/s44319-026-00806-4)
Supplement: Supplementary file 2 — Peer Review File [file 44319_2026_806_MOESM2_ESM.pdf]

EMBO reports

Proximity proteomics reveals a co-evolved LRRK2-regulatory network linked to centrosomes

Marita Eckert, Pasquale Miglionico, Francesca Izzi, Natalia De Oliveira Rosa, Benjamin Riebenbauer, Marius Ueffing, Francesco Raimondi, and Christian Gloeckner

Corresponding author(s): Christian Gloeckner (Johannes.Gloeckner@dzne.de) , Francesco Raimondi (francesco.raimondi@sns.it)

Review Timeline:

Submission Date:	10th Oct 24
Editorial Decision:	11th Nov 24
Revision Received:	5th Sep 25
Editorial Decision:	12th Nov 25
Revision Received:	1st Apr 26
Accepted:	29th Apr 26

Editor: Martina Rembold

Transaction Report:

(Note: With the exception of the correction of typographical or spelling errors that could be a source of ambiguity, letters and reports are not edited. Depending on transfer agreements, referee reports obtained elsewhere may or may not be included in this compilation. Referee reports are anonymous unless the Referee chooses to sign their reports.)

1st Editorial Decision **11th Nov 2024**

Dear Dr. Gloeckner

Thank you for the submission of your research manuscript to our journal. We have now received the full set of referee reports that is copied below.

As you will see, the referees acknowledge that the findings are interesting and overall acknowledge the resource value of your data, but they also raise a number of concerns and have suggestions how to further strengthen the data that should be addressed. All referees comment on the analysis shown in Figure 3F, the effect of CYLD on LRRK2 and these data and conclusions need to be strengthened. Please also add further validation of potential interactors

Given these constructive comments, we would like to invite you to revise your manuscript with the understanding that the referee concerns (as detailed above and in their reports) must be fully addressed and their suggestions taken on board. Please address all referee concerns in a complete point-by-point response. Acceptance of the manuscript will depend on a positive outcome of a second round of review. It is EMBO Reports policy to allow a single round of revision only and acceptance or rejection of the manuscript will therefore depend on the completeness of your responses included in the next, final version of the manuscript.

We realize that it is difficult to revise to a specific deadline. In the interest of protecting the conceptual advance provided by the work, we recommend a revision within 3 months (February 11th). Please discuss the revision progress ahead of this time with the editor if you require more time to complete the revisions.

I am also happy to discuss the revision further via e-mail or a video call, if you wish.

A few points to consider before resubmitting the revised manuscript:
- Your study will be published in our resource section. For resource articles we encourage you to describe relevant parts of the methods using a step-by-step protocol format with bullet points, to facilitate the adoption of the methodologies across labs. Please include a Reagents and Tools table (see point 12 below).
- Please move the Data availability section to the end of the Methods section and please insert links that resolve directly to the datasets (not just to the database, see point 7 below).
- Supplementary tables S1 and S2-S6 should be renamed to Dataset EV#. They need a legend in a separate tab of the .xls sheet. The legends for these should be removed from the manuscript text.
- We recommend depositing computer code to a public repository, e.g. Gitub, and adding this information to the Data availability section (see point 7).

*****IMPORTANT NOTE:
We perform an initial quality control of all revised manuscripts before re-review. Your manuscript will FAIL this control and the handling will be delayed IN CASE the following APPLIES:

1) A data availability section providing access to data deposited in public databases is missing. If you have not deposited any data, please add a sentence to the data availability section that explains that.

2) Your manuscript contains statistics and error bars based on n=2. Please use scatter blots in these cases. No statistics should be calculated if n=2.

When submitting your revised manuscript, please carefully review the instructions that follow below. Failure to include requested items will delay the evaluation of your revision.*****

When submitting your revised manuscript, we will require:

1) a .docx formatted version of the manuscript text (including legends for main figures, EV figures and tables). Please make sure that the changes are highlighted to be clearly visible.

2) individual production quality figure files as .eps, .tif, .jpg (one file per figure).
Please download our Figure Preparation Guidelines (figure preparation pdf) from our Author Guidelines pages https://www.embopress.org/page/journal/14693178/authorguide for more info on how to prepare your figures.

3) a .docx formatted letter INCLUDING the reviewers' reports and your detailed point-by-point responses to their comments. As part of the EMBO Press transparent editorial process, the point-by-point response is part of the Review Process File (RPF), which will be published alongside your paper.

4) a complete author checklist, which you can download from our author guidelines (<https://www.embopress.org/page/journal/14693178/authorguide>). Please insert information in the checklist that is also reflected in the manuscript. The completed author checklist will also be part of the RPF.

5) Please note that all corresponding authors are required to supply an ORCID ID for their name upon submission of a revised manuscript (<https://orcid.org/>). Please find instructions on how to link your ORCID ID to your account in our manuscript tracking system in our Author guidelines (<https://www.embopress.org/page/journal/14693178/authorguide#authorshipguidelines>)

6) We replaced Supplementary Information with Expanded View (EV) Figures and Tables that are collapsible/expandable online. A maximum of 5 EV Figures can be typeset. EV Figures should be cited as 'Figure EV1, Figure EV2" etc... in the text and their respective legends should be included in the main text after the legends of regular figures.

- For the figures that you do NOT wish to display as Expanded View figures, they should be bundled together with their legends in a single PDF file called *Appendix*, which should start with a short Table of Content. Appendix figures should be referred to in the main text as: "Appendix Figure S1, Appendix Figure S2" etc. See detailed instructions regarding expanded view here: <https://www.embopress.org/page/journal/14693178/authorguide#expandedview>

- Additional Tables/Datasets should be labeled and referred to as Table EV1, Dataset EV1, etc. Legends have to be provided in a separate tab in case of .xls files. Alternatively, the legend can be supplied as a separate text file (README) and zipped together with the Table/Dataset file.

7) Before submitting your revision, primary datasets (and computer code, where appropriate) produced in this study need to be deposited in an appropriate public database (see < https://www.embopress.org/page/journal/14693178/authorguide#dataavailability>).

The accession numbers and database should be listed in a formal "Data Availability " section (placed after Materials & Method) that follows the model below (see also < https://www.embopress.org/page/journal/14693178/authorguide#dataavailability>). Please note that the Data Availability Section is restricted to new primary data that are part of this study.

Data availability

The datasets (and computer code) produced in this study are available in the following databases:

- RNA-Seq data: Gene Expression Omnibus GSE46843 (https://www.ncbi.nlm.nih.gov/geo/query/acc.cgi?acc=GSE46843)
- [data type]: [name of the resource] [accession number/identifier/doi] ([URL or identifiers.org/DATABASE:ACCESSION])

*** Note - All links should resolve to a page where the data can be accessed. ***

8) At EMBO Press we ask authors to provide source data for the main figures. Our source data coordinator will contact you to discuss which figure panels we would need source data for and will also provide you with helpful tips on how to upload and organize the files.

Additional information on source data and instruction on how to label the files are available <https://www.embopress.org/page/journal/14693178/authorguide#sourcedata>.

9) The journal requires a statement specifying whether or not authors have competing interests (defined as all potential or actual interests that could be perceived to influence the presentation or interpretation of an article). In case of competing interests, this must be specified in your disclosure statement. Further information: https://www.embopress.org/competing-interests

10) Figure legends and data quantification:
The following points must be specified in each figure legend:

- the name of the statistical test used to generate error bars and P values,
- the number (n) of independent experiments (please specify technical or biological replicates) underlying each data point,
- the nature of the bars and error bars (s.d., s.e.m.)

- If the data are obtained from n {less than or equal to} 5, show the individual data points in addition to the SD or SEM.
- If the data are obtained from n {less than or equal to} 2, use scatter blots showing the individual data points.

Discussion of statistical methodology can be reported in the materials and methods section, but figure legends should contain a basic description of n, P and the test applied.

See also the guidelines for figure legend preparation:

https://www.embopress.org/page/journal/14693178/authorguide#figureformat

- Please also include scale bars in all microscopy images and define their size in the legend, not the image.

11) Our journal encourages inclusion of *data citations in the reference list* to directly cite datasets that were re-used and obtained from public databases. Data citations in the article text are distinct from normal bibliographical citations and should directly link to the database records from which the data can be accessed. In the main text, data citations are formatted as follows: "Data ref: Smith et al, 2001" or "Data ref: NCBI Sequence Read Archive PRJNA342805, 2017". In the Reference list, data citations must be labeled with "[DATASET]". A data reference must provide the database name, accession number/identifiers and a resolvable link to the landing page from which the data can be accessed at the end of the reference. Further instructions are available at <https://www.embopress.org/page/journal/14693178/authorguide#referencesformat>.

12) All Materials and Methods need to be described in the main text using our 'Structured Methods' format. According to this format, the Methods section includes a Reagents and Tools Table (listing key reagents, experimental models, software and relevant equipment and including their sources and relevant identifiers) followed by a Methods and Protocols section describing the methods, ideally using a step-by-step protocol format. The aim is to facilitate adoption of the methodologies across labs. Please download and fill our Reagents and Tools Table template (.docx), which you can find in our author guidelines: https://www.embopress.org/page/journal/14693178/authorguide#structuredmethods.
When submitting your revised manuscript, please do not include the Reagents and Tools Table in the Methods section of the manuscript but upload it as a separate file choosing the file type "Reagent Table".
An example of a Method paper with Structured Methods can be found here: https://www.embopress.org/doi/10.15252/msb.20178071.

13) As part of the EMBO publication's Transparent Editorial Process, EMBO Reports publishes online a Review Process File to accompany accepted manuscripts. This File will be published in conjunction with your paper and will include the referee reports, your point-by-point response and all pertinent correspondence relating to the manuscript.

You are able to opt out of this by letting the editorial office know (emboreports@embo.org). If you do opt out, the Review Process File link will point to the following statement: "No Review Process File is available with this article, as the authors have chosen not to make the review process public in this case."

We would also welcome the submission of cover suggestions, or motifs to be used by our Graphics Illustrator in designing a cover.

I look forward to seeing a revised form of your manuscript when it is ready and please let me know if you have questions or comments regarding the revision.

Yours sincerely,

Martina Rembold, PhD
Senior Editor
EMBO reports

====================

Referee #1:

Eckert and Miglionico et al. use a variety of proximity-dependent biotinylation approaches to probe the interactome of LRRK2. They couple their interactomes with a co-evolutionary analysis and structural modeling using AlphaFold-multimer. This analysis coupled with LRRK2 interactomes in type I kinase inhibitor conditions or with Rab29 overexpression, lead the authors to conclude that different LRRK2 interactors engage with LRRK2 in different conformational states and in different subcellular compartments. A bit more validation of a few interactors would strengthen the conclusions of the current manuscript.

Major Points:
(1) For Figure 3F -

a. why was the Y1699C variant used instead of the WT (matching the proteomics)?
b. The authors should quantify three biological/technical replicates.
c. Is the expression of ubiquitin required to drive the increased stability of LRRK2?

(2) Why are the LRR-Roc-Cor-Kinase domains all categorized together for "core" interactors? They are completely different functional domains.

(3) For the manuscript to be more, authors should determine the co-evolutionary network for LRRK2 interactors in Rab29 O/E conditions

(4) The author should discuss the advantages and disadvantages of using 293 cells as a model cell for LRRK2, especially since there is a marked increase in microtubule-dependent binding of LRRK2 in 293s, which is not seen in other cell types. This might explain why many of their GO biological processes were enriched for microtubule- and mitotic- proteins.

(5) Secondary verification should be performed (with co-immunoprecipitation or other methods), especially for those interactors that are predicted to be more stable such as CEP85 and CYLD.

Minor Points:

(1) Some figures (including Figure 3 and Figure 6) need to be higher quality and more legible fonts/labels because it is hard to read.

(2) Bonet-Ponce et al., 2020 used proximity dependent labeling with APEX. Please cite appropriately.

(3) Labeling the model structures all figures including in Figure 4B and 5C-E will be very helpful to the reader.

Referee #2:

The manuscript by Eckert et al. presents results from a well-controlled and thorough BioID proximity proteome analysis of wildtype LRRK2. The authors reveal novel interactors and identify structural and functional modules enriched for components related to centrosome and microtubules, accompanied by structural modeling of binary interactions. In addition, they use type I kinase inhibitor or co-expression with Rab29 to probe for the proximity proteome of the active conformational state of LRRK2, which reveals proteins associated with centriolar satellites.

Minor comments below:
1. Page 5: the authors stipulate that cells were incubated with biotin in serum-free conditions for up to 16 h, or were incubated with biotin after overnight starvation (I assume this is serum-free medium too). Why did the authors opt for such overnight starvation? Serum-starvation will induce autophagy as well as ciliogenesis. How may this affect the localization of LRRK2 and hence its proximity proteome? This should be acknowledged and discussed.

2. Page 13 and Figure 3F: the authors employ Y1699C to show that co-expression with CYLD alters LRRK2 stability. They should perform the same experiments with wt LRRK2, and show quantification over several experiments to demonstrate significance.

3. Page 20: they mention that LRRK2 inhibition with type I inhibitors induces its destabilization and degradation. No reference is provided, and these findings have not been reproduced in all cases. Please rephrase.

4. The authors may want to acknowledge limitations of their study, such as the use of LRRK2 overexpression, which may lead to erroneous interactions not normally occurring in the context of endogenous LRRK2.

Referee #3:

The authors have performed proximity proteomics to identify new interactors of LRRK2.

The topic (identification of interactors of LRRK2, a protein that plays a central role in PD) is relevant. The experiments are methodologically sound and mostly seem to have been performed carefully. The identified networks of LRRK2 proximity partners are valuable information. However, this work does not provide substantial novel insights into the interactions or functions of LRRK2. Previous work has already shown that LRRK2 interacts with cytoskeletal components linked to the centrosome and microtubules.

Other comments:
- Fig. 3F: the experiment about the effect of CYLD on LRRK2 is a bit superficial. Quantification of the effect of CYLD on LRRK2 Y1688C levels is missing. Does CYLD coexpression also increase the level of wild-type LRRK2? Does CYLD bind to LRRK2?

The authors propose that CYLD stabilizes Y1669C LRRK2 by deubiquitinating K63 ubiquitin chains. Is the effect of CYLD on Y1669C LRRK2 abolished when expressing K63R ubiquitin (which cannot form K63 chains but is otherwise identical to wild-type ubiquitin)? Which conditions do they mean exactly in the confusing sentence (Line 372): "The condition without CYLD co-expression showed overall higher LRRK2 levels compared to the HA-WT ubiquitin condition."? The conditions without CYLD overexpression in Fig. 3F (lane 2 and lane 4) actually show lower LRRK2 levels than the other lanes.

- The authors mention several times that proximity-based methods are much more sensitive than affinity-based methods for detection of dynamic or transient interactions. How do they explain that they hardly detected any of the many Rab regulators/substrates of LRRK2? They only detected Rab8a with LRRK2-miniTurbo when coexpressing Rab29.
- The fact that the experiments were conducted exclusively in the cell line HEK293T should be discussed as a limitation.
- P. 5, line 109-110: "In case of the mini turbo tag, cells have been incubated with 50 uM biotin after o/n starvation for 2h." Please clarify what is meant by o/n starvation for 2h.
- P. 10: a figure with schematic drawings of the different used constructs would be helpful.
- P. 5, line 274-275: "a tag-only control". For the sake of clarity please specify whether the HA-tag or the TurboID tag is meant here.
- The labels in some of the figures (e.g. Fig. 2A, Fig. 3A-B-D) are very small.
- Suppl. Fig. 1: in the LRRK2-BioID1 and -BioID2 conditions there is much less p-Rab10 than in the LRRK2-miniTurbo condition. Why?
- Suppl. Fig. 1B: what is BirA LRRK2? Is this a different construct than LRRK2-BioID1? What is PEI?

Point-to-point response:

Rebuttal to Reviewers' Comments – EMBOR-2024-60555-T
Manuscript Title: "Proximity proteomics reveals a co-evolved LRRK2-regulatory network linked to centrosomes"

Referee #1:

Eckert and Miglionico et al. use a variety of proximity-dependent biotinylation approaches to probe the interactome of LRRK2. They couple their interactomes with a co-evolutionary analysis and structural modeling using AlphaFold-multimer. This analysis coupled with LRRK2 interactomes in type I kinase inhibitor conditions or with Rab29 overexpression, lead the authors to conclude that different LRRK2 interactors engage with LRRK2 in different conformational states and in different subcellular compartments. A bit more validation of a few interactors would strengthen the conclusions of the current manuscript.

Major Points:

(1) For Figure 3F -

a. why was the Y1699C variant used instead of the WT (matching the proteomics)?

b. The authors should quantify three biological/technical replicates.

c. Is the expression of ubiquitin required to drive the increased stability of LRRK2?

Response: *We have fully revised this experiment. The updated Figure 3G–H now includes both wild-type and Y1699C LRRK2, multiple replicates, and quantitative capillary Western blot analysis. Also see lines 399ff in the text.*

(2) Why are the LRR-Roc-Cor-Kinase domains all categorized together for "core" interactors? They are completely different functional domains.

Response: *We thank the reviewer for this important point. We referred to these domains as "Core" because, from an evolutionary perspective, they constitute the most conserved regions of the LRRK2 protein. The rationale for this terminology is explained in lines (line 446ff).*

(3) For the manuscript to be more, authors should determine the co-evolutionary network for LRRK2 interactors in Rab29 O/E conditions

Response: *We thank the reviewer for suggestion. The co-evolutionary and structural analysis of the interactome set obtained with Rab29 overexpression, as well as of the one obtained with MLi2 treatment, were already included in the first version of the manuscript, specifically in Figure 6F and Figure 7. In particular, the RAB29 interactome appears to have similar co-evolution distributions with respect to the APO state, while the MLi2 interactome has a significantly higher co-evolution (Figure 6F).*

(4) The author should discuss the advantages and disadvantages of using 293 cells as a model cell for LRRK2, especially since there is a marked increase in microtubule-dependent binding of LRRK2 in 293s, which is not seen in other cell types. This might explain why many of their GO biological processes were enriched for microtubule- and mitotic- proteins.

Response: *We have added an extra section discussing the limitations of the study (line 685ff).*

(5) Secondary verification should be performed (with co-immunoprecipitation or other methods), especially for those interactors that are predicted to be more stable such as CEP85 and CYLD.

Response: *We thank the reviewer for this important suggestion. For CYLD, we now provide robust functional validation, including validation of the mass spectrometric data by Western Blot and stability assays, demonstrating its effect on both wild-type and mutant LRRK2 (line 399ff). In addition, we have expanded our data on centriolar satellite–associated regulation of LRRK2. Specifically, we show that siRNA-mediated knockdown of PCM1, the central scaffold of centriolar satellites, abolishes proximity interaction with SSX2IP, a satellite protein enriched in the LRRK2 interactome upon MLi-2 treatment. These data provide additional support for a functional role of the centriolar satellite compartment in modulating LRRK2 interaction dynamics (see: figures 3+6 and supplemental figures S5+S6; line 508ff).*

Minor Points:

(1) Some figures (including Figure 3 and Figure 6) need to be higher quality and more legible fonts/labels because it is hard to read.

Response: *We have updated all figures, including Figures 3 and 6, to ensure improved resolution, enlarged fonts, and enhanced label clarity. All figure panels have been regenerated at publication-quality standards to guarantee readability in both print and digital formats.*

(2) Bonet-Ponce et al., 2020 used proximity dependent labeling with APEX. Please cite appropriately.

Response: *We have now appropriately cited Bonet-Ponce et al., 2020 in the Discussion section of the manuscript when discussing proximity labeling approaches, and we highlight their use of APEX in the context of LRRK2 pathway analysis (line 581f).*

(3) Labeling the model structures all figures including in Figure 4B and 5C-E will be very helpful to the reader.

Response: *We have now labelled the domains and interaction partners of all the 3D structures in figures 4, 5 and 7.*

Referee #2:

The manuscript by Eckert et al. presents results from a well-controlled and thorough BioID proximity proteome analysis of wildtype LRRK2. The authors reveal novel interactors and identify structural and functional modules enriched for components related to centrosome and microtubules, accompanied by structural modeling of binary interactions. In addition, they use type I kinase inhibitor or co-expression with Rab29 to probe for the proximity proteome of the active conformational state of LRRK2, which reveals proteins associated with centriolar satellites.

Minor comments below:

1. Page 5: the authors stipulate that cells were incubated with biotin in serum-free conditions for up to 16 h, or were incubated with biotin after overnight starvation (I assume this is serum-free medium too). Why did the authors opt for such overnight starvation? Serum-starvation will induce autophagy

as well as ciliogenesis. How may this affect the localization of LRRK2 and hence its proximity proteome? This should be acknowledged and discussed.

Response: *We thank the reviewer for this important point. Starvation was used to synchronize the cells and, in light of the physiological functions of LRRK2, to enrich for post-mitotic states in which its centrosomal and ciliary roles are more prominent. We now clarify this in the revised manuscript (lines 110ff).*

2. Page 13 and Figure 3F: the authors employ Y1699C to show that co-expression with CYLD alters LRRK2 stability. They should perform the same experiments with wt LRRK2, and show quantification over several experiments to demonstrate significance.

Response: *We have added quantitative data using a capillary Western approach. Also see response to Referee 1, point 1.*

3. Page 20: they mention that LRRK2 inhibition with type I inhibitors induces its destabilization and degradation. No reference is provided, and these findings have not been reproduced in all cases. Please rephrase.

Response: *The reference has been added (Lobbestael et al., 2016; lines 660f).*

4. The authors may want to acknowledge limitations of their study, such as the use of LRRK2 overexpression, which may lead to erroneous interactions not normally occurring in the context of endogenous LRRK2.

Response: *We have added an extra section discussing the limitations of the study. Also see response to Referee 1, point 4.*

Referee #3:

The authors have performed proximity proteomics to identify new interactors of LRRK2.

The topic (identification of interactors of LRRK2, a protein that plays a central role in PD) is relevant. The experiments are methodologically sound and mostly seem to have been performed carefully. The identified networks of LRRK2 proximity partners are valuable information. However, this work does not provide substantial novel insights into the interactions or functions of LRRK2. Previous work has already shown that LRRK2 interacts with cytoskeletal components linked to the centrosome and microtubules.

Other comments:

- Fig. 3F: the experiment about the effect of CYLD on LRRK2 is a bit superficial. Quantification of the effect of CYLD on LRRK2 Y1688C levels is missing. Does CYLD coexpression also increase the level of wild-type LRRK2? Does CYLD bind to LRRK2? The authors propose that CYLD stabilizes Y1669C LRRK2 by deubiquitinating K63 ubiquitin chains. Is the effect of CYLD on Y1669C LRRK2 abolished when expressing K63R ubiquitin (which cannot form K63 chains but is otherwise identical to wild-type ubiquitin)? Which conditions do they mean exactly in the confusing sentence (Line 372): "The condition without CYLD co-expression showed overall higher LRRK2 levels compared to the HA-WT ubiquitin condition."? The conditions without CYLD overexpression in Fig. 3F (lane 2 and lane 4) actually show lower LRRK2 levels than the other lanes.

Response: *We thank the reviewer for this thorough and constructive set of observations. We have addressed each point as follows:*

(1) Starvation was used to synchronize the cells and, in light of the physiological functions of LRRK2, to enrich for post-mitotic states in which its centrosomal and ciliary roles are more prominent. (line 110 ff; also see point 1/ reviewer 2).

(2) Quantification and Wild-Type LRRK2: We have now included quantification data from three biological replicates for both wild-type and Y1699C LRRK2, confirming that CYLD increases the levels of both forms. These data are included in the revised Figure 3F and accompanying source data.

(3) CYLD–LRRK2 Binding: We now provide additional functional validation for CYLD, including confirmation of the mass spectrometry data by western blot (BioID) and protein stability assays, demonstrating its stabilizing effect on both wild-type and Y1699C-mutant LRRK2 (also see comment 5 of Referee 1). To assess the specificity of this effect, we used a K63-only ubiquitin construct in which all lysine residues except K63 are mutated to arginine—thus permitting polymerization exclusively via lysine 63. This construct was chosen based on CYLD's established specificity for K63-linked polyubiquitin chains (lines 399ff).

(4) Clarification of Sentence (line 372 in the original manuscript): We thank the reviewer for pointing out the unclear phrasing. In the course of incorporating new quantitative data, we have substantially revised and expanded the entire section (beginning at line 399). The revised text now provides a clearer description of the experimental results and resolves the inconsistency noted in the original sentence.

- The authors mention several times that proximity-based methods are much more sensitive than affinity-based methods for detection of dynamic or transient interactions. How do they explain that they hardly detected any of the many Rab regulators/substrates of LRRK2? They only detected Rab8a with LRRK2-miniTurbo when coexpressing Rab29.

Response: *We agree this is an important point. The limited detection of Rab GTPases in our BioID dataset may reflect several contributing factors. Although BioID is generally well suited to capture dynamic and transient interactions, Rab proteins are small and contain relatively few accessible lysine residues, which may reduce biotinylation efficiency compared to larger, more modifiable proteins. Additionally, LRRK2 is known to phosphorylate membrane-bound Rabs, and under physiological conditions, only a small fraction (<1%) of total Rab proteins are phosphorylated. This low abundance of active, membrane-associated Rab substrates may fall below the sensitivity threshold of proximity labeling approaches or may be insufficiently extracted during lysis and enrichment. Together, these factors likely contribute to the underrepresentation of Rabs in our proximity proteomic dataset. We also addressed this in the discussion of the revised manuscript (line 578ff).*

- The fact that the experiments were conducted exclusively in the cell line HEK293T should be discussed as a limitation.

Response: *We have added an extra section discussing the limitations of the study. Also see response to Referee 1, point 4.*

- P. 5, line 109-110: "In case of the mini turbo tag, cells have been incubated with 50 uM biotin after o/n starvation for 2h." Please clarify what is meant by o/n starvation for 2h.

Response: *We have fixed ambiguous phrasing. The biotin incubation for 2h was following the o/n starvation (line 110).*

- P. 10: a figure with schematic drawings of the different used constructs would be helpful.

Response: *For a better overview, a list of all constructs is given in an extra document ('Reagents and Tools' table).*

- P. 5, line 274-275: "a tag-only control". For the sake of clarity please specify whether the HA-tag or the TurboID tag is meant here.

Response: *We apologize for this unclarity. The BioID tag was used as reference, as a homogeneous distribution within the cytosol can be expected. This is also indicated by initial experiments with IHC. We have clarified this in the revised manuscript.*

- The labels in some of the figures (e.g. Fig. 2A, Fig. 3A-B-D) are very small.

Response: *We have updated all figures, including Figures 3 and 6, to ensure improved resolution, enlarged fonts, and enhanced label clarity. All figure panels have been regenerated at publication-quality standards to guarantee readability in both print and digital formats.*

- Suppl. Fig. 1: in the LRRK2-BioID1 and -BioID2 conditions there is much less p-Rab10 than in the LRRK2-miniTurbo condition. Why?

Response: *We thank the reviewer for this observation. Indeed, in the first experiment (panel A), which includes the inhibitor control, the miniTurbo (mT)-LRRK2 fusion construct exhibited a higher Rab10-pT73 signal compared to the BioID1 and BioID2 fusions. However, in a replication experiment (panel B), all tested LRRK2 constructs—including BioID1, BioID2, and mT—showed similarly low basal Rab10-pT73 levels upon transient transfection. The stable mT-LRRK2 cell line displayed consistently higher Rab10-pT73 levels in both experiments, most likely due to differences in transfection efficiency under transient conditions, where not all cells express LRRK2 at sufficient levels. Importantly, our primary objective was to assess whether the fusion tag alters LRRK2 kinase activity. This concern is addressed in panel B, which includes all constructs—alongside an SF-TAP-LRRK2 WT control—and shows comparable Rab10-pT73 levels across conditions, indicating that none of the tested tags interfere with LRRK2-mediated phosphorylation.*

- Suppl. Fig. 1B: what is BirA LRRK2? Is this a different construct than LRRK2-BioID1? What is PEI?

Response: *We thank the reviewer for pointing this out. The terms BirA and BioID1 were used synonymously in earlier versions, and we have now corrected the figure labeling for consistency. PEI stands for polyethylenimine, which is used as a transfection reagent. We have updated the manuscript to include the full term upon first mention.*

We thank the reviewers for their thoughtful and constructive feedback. We trust that the revised manuscript and additional experiments fully address the concerns raised and significantly strengthen our work.

Sincerely,
The Authors

Dear Dr. Gloeckner

Thank you for the submission of your revised manuscript to EMBO Reports. I sincerely apolgoize for the delay in handling it. We have received the reports from the two referees who were asked to assess it (copied below). As you will see, referee #1 considers his/her concerns adequately addressed, but referee #3 has a number of concerns related to the new data added during the revision.

Referee #3 is not fully convinced by the new data on the interaction of LRRK2 with CYLD. The referee considers the effects minor and only visible if K63-Ub is co-expressed. The referee further points out that the quantification is based on technical replicates. I note that you have two independent experiments, but show them separately (one in 3H, the other on in S5C). I think that the remaining concerns from Referee #3 should be addressed in a second round of revision, also given that this concern had been raised in the first round of review, and that I emphasized that the effect of CYLD on LRRK2 needed to be strengthened when inviting revision.

From the editorial side, there are also a few things that need to be addressed, as follows:

- Please remove all figures from the manuscript text. Only the main and EV figure legends should be kept at the end of the manuscript.

- Please provide up to 5 keywords on the title page.

- Please provide a 'Disclosure and competing interests statement'. For more information see https://www.embopress.org/page/journal/14693178/authorguide#conflictsofinterest

- Regarding the Author Contributions, we now use CRediT to specify the contributions of each author in the journal submission system. Therefore, please remove the Author Contributions from the manuscript file and make sure that the author contributions in our online manuscript tracking system are correct and up-to-date. The information you specified in the system will be automatically retrieved and typeset into the article. You can enter additional information in the free text box provided, if you wish. See also our guide to authors https://www.embopress.org/page/journal/14693178/authorguide#authorshipguidelines.

- References: et al needs to be used after 10 author names; DOIs should only be used for preprints and datasets that have not been published yet. The section heading should be corrected to References.

- Raig et al 2024, Evans et al 2021, Guaitoli et al 2023, and Miglionico et al 2024 are preprints. In the text it should be cited as (preprint: AUTHOR NAME et al, year) and in the reference list you should add [PREPRINT] at the end of the reference.

- Author Checklist: please add the manuscript ID# but missing md ID# in line 6.

- Please add the following funding information/list them as separte funder in the online manuscript tracking system: grant MJFF-023920 (for The Michael J. Fox Foundation for Parkinson's Research) and iMed - the Helmholtz Initiative on Personalized Medicine.

- Please provide callouts for Figure 7B, C.

- Tables EV1 and EV2 should be updated to Dataset EV1 and Dataset EV2 in all places (source file names, table legends, titles in the system, manuscript callouts); their legends should be removed from the manuscript text.

- The Appendix Figures and their legends need to be removed from the manuscript file and instead be provided as a single Appendix PDF file. The Appendix PDF needs a table of content with page numbers on the title page.

- The manuscript sections should be in the following order: Title page - Abstract & Keywords - Introduction - Results - Discussion - Methods - Data Availability - Acknowledgments - Disclosure Statement & Competing Interests - References - Figure Legends - (Main Tables with legends if applicable) - Expanded View Figure Legends.

- Please address the following points in the figure legends and please make sure to always define the nature of the replicates (technical, biological, independent):
a) Please define the annotated p values ****/***/**/* as well as provide the exact p-values for the same in the legend of figure S5 C as appropriate.
b) Please provide the exact p values in the legends of figures 3A, H; 4D, E; 5E-G; 6F, 7D, unless $p<0.0001$.
c) Please indicate the statistical test used for data analysis in the legend of figure S6 B
d) Please note that information related to n is missing in the legends of figures 3A, 4D, E; 5E-G; 6F, 7D, E; S5 C, S6 B
d) Please note that the error bars are not defined in the legends of figures 3H, S5 A

- Please describe your findings in the Abstract in present tense.

- Finally, EMBO Reports papers are accompanied online by
A) a short (1-2 sentences) summary of the findings and their significance,
B) 2-3 bullet points highlighting key results and
C) a schematic summary figure that provides a sketch of the major findings (not a data image).
Please provide the summary figure as a separate file in PNG or JPG format at a size of 550x300-600 pixels (width x height). Please note that the size is rather small and that text needs to be readable at the final size. Please send us this information along with the revised manuscript.

I look forward to seeing a revised form of your manuscript when it is ready.

Yours sincerely,

Martina Rembold, PhD
Senior Editor
EMBO reports

====================

Referee #1:

The authors adequately addressed my concerns.

There are quite a few issues with grammar throughout the manuscript (as an example: Lines 581-582 "For LRRK2, the first APEX2
582 dataset only recently became available recently") and too much detail in certain places (as an example: the authors do not need to state in the results section that Gateway Cloning was used).

Another minor concern: When discussing the limitations of the study, my initial concern is that 293s has more observable microtubule-binding then other cell types (compared to astrocytes for example). This needs to be addressed as a limitation because it could certainly be why more centrosomal proteins and MT proteins are present in BioID datasets. Also, there is a wealth of evidence that LRRK2 functions outside of the "neuronal context", so this does not need to be brought up as a limitation.

Referee #3:

1. The novelty of the work is still somewhat limited, as previous work already shown that LRRK2 interacts with cytoskeletal components linked to the centrosome and microtubules.

2. The new experiments regarding the interaction of LRRK2 with CYLD (Fig. 3G-H and Fig. S5) are not convincing, for several reasons:
- The text (p. 14) states: "Notably, co-expression of CYLD let to a marked increase in LRRK2 stability. This effect was observed for both wild-type LRRK2 and the pathogenic Y1999C variant" and "In summary, ...CYLD co-expression enhances LRRK2 protein stability". However, Fig. 3H and Fig. S5C do not show a significant effect of CYLD overexpression on protein levels of either wild-type or Y1699C LRRK2. So the statements in the text are not supported by the data.
- Whether CYLD overexpression has any effect on LRRK2 protein levels in the absence of ubiquitin overexpression is unclear.
- The graph in Fig. 3H is based on 3 technical replicates (the same biological sample was analyzed three times). The graph in Fig. S5C is also based on 3 technical replicates of a second biological replicate. To make the data more convincing, at least 3 biological replicates should be performed. Technical replicates of the same biological sample should not be presented as independent data points.

Point-by-point response to reviewers

Manuscript: EMBOR-2024-60555V3

We thank the editor and the reviewers for their careful and constructive assessment of our revised manuscript. We have addressed all comments raised by the referees and the editorial office and revised the manuscript accordingly. In particular, we substantially strengthened the CYLD-related data and revised their interpretation to address the concerns raised by Referee #3. We have also implemented all requested editorial changes.

Referee #1

We thank the referee for the positive evaluation and for noting that the main concerns had been adequately addressed.

Comment 1: There are quite a few issues with grammar throughout the manuscript.

Response: We carefully revised the manuscript for grammar and language throughout to improve clarity and readability.

Comment 2: Too much methodological detail in certain places (e.g. Gateway cloning in Results).

Response: We agree and streamlined the Results section by removing unnecessary methodological detail.

Comment 3: HEK293 cells show increased microtubule binding; this should be discussed as a limitation.

Response: We thank the referee for this important point. We now explicitly address this in the "Limitations of the study" section. We state that HEK293T cells may exhibit cell-type-specific biases, including increased representation of microtubule-binding proteins, which could contribute to the enrichment of centrosomal and microtubule-associated proteins in the BioID datasets.

Comment 4: LRRK2 function outside neuronal context does not need to be presented as a limitation.

Response: We agree and revised the text accordingly. References to this point as a limitation were removed.

Referee #3

We thank the referee for the careful and critical evaluation of the CYLD-related data. We substantially revised both the experimental data and their interpretation to address these concerns.

Comment 1: The novelty of the work is still somewhat limited, as previous work already showed that LRRK2 interacts with cytoskeletal components linked to the centrosome and microtubules.

Response: We agree that previous studies have linked LRRK2 to cytoskeletal and centrosomal components. However, our study extends these observations in several important ways.

First, *using co-evolutionary analysis of the proximity proteomics interactome, we identify a co-evolved subset of LRRK2 interaction partners enriched in centrosomal and cytoskeletal proteins, suggesting that these LRRK2-associated components are evolutionarily conserved.*

Second, *we show that the LRRK2 interactome is state dependent, as distinct interaction profiles are observed upon MLi-2 treatment or RAB29 co-expression, linking LRRK2 activity and localization to specific interaction patterns.*

Third, *by incorporating AlphaFold-based structural modeling, we stratify interactions according to conformational state and predicted interface usage, thereby distinguishing partners associated with different structural states and domains of the LRRK2 architecture.*

Together, *this integrative approach provides a system-level view of LRRK2 interaction organization and helps contextualize how previously described cytoskeletal associations may be organized and regulated. We revised the manuscript to better emphasize these points.*

<u>Comment 2:</u> The new experiments regarding the interaction of LRRK2 with CYLD are not convincing, for several reasons:

The statements in the text are not supported by the data.

Whether CYLD overexpression has any effect on LRRK2 protein levels in the absence of ubiquitin overexpression is unclear.

The quantification is based on technical replicates rather than biological replicates.

<u>Response:</u> We directly addressed these concerns by substantially revising both the experimental data and their interpretation.

First, *we now explicitly show that the increase in LRRK2 levels upon CYLD co-expression is also observed under basal conditions, that is, in the absence of ubiquitin overexpression (Figure 3G; Appendix Figure S5A). This directly addresses the concern that the observed effect might depend on ubiquitin overexpression. This experiment was performed twice, with N = 6 biological replicates in each independent experiment.*

Second, *we repeated the experiments with co-expression of wild-type ubiquitin and K63-only ubiquitin shown in the original version of the manuscript (Appendix Figure S5B). These experiments reproduced the observed trend. However, to avoid overinterpretation, we now explicitly state that these data do not allow firm conclusions regarding ubiquitin linkage specificity. These experiments were performed with N = 3 biological replicates.*

Third, *we fully agree that biological replicates are required. We therefore repeated the experiments and updated all quantifications accordingly. Quantifications are now based on independent biological replicates, and technical and biological replicates are clearly distinguished throughout the manuscript and in the figure legends (see point 1 and 2).*

Together, *we believe that these revisions substantially strengthen the robustness, transparency, and interpretation of the CYLD-related findings.*

Additional changes

In addition to addressing the reviewer comments, we implemented all editorial requests, including:

- *revision of manuscript structure and section order;*
- *addition of keywords, summary text, key results, and a schematic summary figure;*
- *inclusion of a Disclosure and competing interests statement;*
- *removal of Author Contributions from the manuscript file and transfer of contribution details to the online submission system;*
- *revision of references and preprint formatting;*
- *updating of Tables EV1 and EV2 to Dataset EV1 and Dataset EV2 throughout;*
- *transfer of Appendix figures and legends to a separate Appendix file;*

- *revision of figure legends to define replicate types, statistical tests, exact P-values, and error bars where appropriate;*
- *revision of the Abstract to describe the findings in present tense.*

We believe that these revisions fully address the concerns raised by the reviewers and the editorial office and have significantly strengthened the manuscript.

2nd Revision - Editorial Decision 29th Apr 2026

Christian Gloeckner
German Center for Neurodegenerative Diseases (DZNE)
Tübingen Site
Otfried-Mueller-Strasse 23
Tübingen 72076
Germany

Dear Dr. Gloeckner,

Thank you for your patience while former referee #3 has assessed the final revision of your manuscript. As you will see from the report below, this referee considers the revision adequate and recommends publication pending a minor textual change, which I have implemented on your behalf. Please see the attached document and please let me know if you wish to further amend the proposed change.

I am therefore very pleased to accept your manuscript for publication in the next available issue of EMBO reports. Thank you for your contribution to our journal.

Your manuscript will be processed for publication by EMBO Press. It will be copy edited and you will receive page proofs prior to publication. Please note that you will be contacted by Springer Nature Author Services to complete licensing and payment information.

You may qualify for financial assistance for your publication charges - either via a Springer Nature fully open access agreement or an EMBO initiative. Check your eligibility: https://link.springer.com/journal/44319/how-to-publish-with-us

Should you be planning a Press Release on your article, please get in contact with embo_production@springernature.com as early as possible in order to coordinate publication and release dates.

If you have any questions, please do not hesitate to contact the Editorial Office. Thank you for your contribution to EMBO Reports.

Yours sincerely,

Martina Rembold, PhD
Senior Editor
EMBO reports

Referee #3:

The authors have adequately responded to my concerns.

One minor comment: the 'Limitations of the study' paragraph (p. 23) should be moved to the Discussion instead of being included in the Methods section.

>>> Please note that it is EMBO Reports policy for the transcript of the editorial process (containing referee reports and your response letter) to be published as an online supplement to each paper. If you do NOT want this, you will need to inform the Editorial Office via email immediately. More information is available here: https://link.springer.com/partners/embo-press/editorial-policies#Peer%20review

EMBO Press Author Checklist

Corresponding Author Name: Gloeckner, Christian Johannes; Raimondi, Francesco
Journal Submitted to: EMBO Reports
Manuscript Number: EMBOR-2024-60555

USEFUL LINKS FOR COMPLETING THIS FORM
The EMBO Journal - Author Guidelines
EMBO Reports - Author Guidelines
Molecular Systems Biology - Author Guidelines
EMBO Molecular Medicine - Author Guidelines

Reporting Checklist for Life Science Articles (updated January 2022)

This checklist is adapted from Materials Design Analysis Reporting (MDAR) Checklist for Authors. MDAR establishes a minimum set of requirements in transparent reporting in the life sciences (see Statement of Task: 10.31222/osf.io/9sm4x). Please follow the journal's guidelines in preparing your manuscript.
Please note that a copy of this checklist will be published alongside your article.

Abridged guidelines for figures
1. Data
The data shown in figures should satisfy the following conditions:

- the data were obtained and processed according to the field's best practice and are presented to reflect the results of the experiments in an accurate and unbiased manner.
- ideally, figure panels should include only measurements that are directly comparable to each other and obtained with the same assay.
- plots include clearly labeled error bars for independent experiments and sample sizes. Unless justified, error bars should not be shown for technical replicates.
- if n<5, the individual data points from each experiment should be plotted. Any statistical test employed should be justified.
- Source Data should be included to report the data underlying figures according to the guidelines set out in the authorship guidelines on Data Presentation.

2. Captions
Each figure caption should contain the following information, for each panel where they are relevant:

- a specification of the experimental system investigated (eg cell line, species name).
- the assay(s) and method(s) used to carry out the reported observations and measurements.
- an explicit mention of the biological and chemical entity(ies) that are being measured.
- an explicit mention of the biological and chemical entity(ies) that are altered/varied/perturbed in a controlled manner.
- the exact sample size (n) for each experimental group/condition, given as a number, not a range;
- a description of the sample collection allowing the reader to understand whether the samples represent technical or biological replicates (including how many animals, litters, cultures, etc.).
- a statement of how many times the experiment shown was independently replicated in the laboratory.
- definitions of statistical methods and measures:
  - common tests, such as t-test (please specify whether paired vs. unpaired), simple χ2 tests, Wilcoxon and Mann-Whitney tests, can be unambiguously identified by name only, but more complex techniques should be described in the methods section;
  - are tests one-sided or two-sided?
  - are there adjustments for multiple comparisons?
  - exact statistical test results, e.g., P values = x but not P values < x;
  - definition of 'center values' as median or average;
  - definition of error bars as s.d. or s.e.m.

Please complete ALL of the questions below.
Select "Not Applicable" only when the requested information is not relevant for your study.

Materials

Newly Created Materials	Information included in the manuscript?	In which section is the information available? (Reagents and Tools Table, Materials and Methods, Figures, Data Availability Section)
New materials and reagents need to be available; do any restrictions apply?	Yes	Material and Methods

Antibodies	Information included in the manuscript?	In which section is the information available? (Reagents and Tools Table, Materials and Methods, Figures, Data Availability Section)
For **antibodies** provide the following information:<br>- Commercial antibodies: RRID (if possible) or supplier name, catalogue number and or/clone number<br>- Non-commercial: RRID or citation	Yes	Reagents and Tools Table

DNA and RNA sequences	Information included in the manuscript?	In which section is the information available? (Reagents and Tools Table, Materials and Methods, Figures, Data Availability Section)
**Short novel DNA or RNA including primers, probes**: provide the sequences.	Yes	Material and Methods

Cell materials	Information included in the manuscript?	In which section is the information available? (Reagents and Tools Table, Materials and Methods, Figures, Data Availability Section)
**Cell lines:** Provide species information, strain. Provide accession number in repository **OR** supplier name, catalog number, clone number, and/**OR** RRID.	Yes	Material and Methods, Reagents and Tools Table
**Primary cultures:** Provide species, strain, sex of origin, genetic modification status.	Not Applicable	
Report if the cell lines were recently **authenticated** (e.g., by STR profiling) and tested for mycoplasma contamination.	Yes	Cell line was authenticated and tested for mycoplasma contamination

Experimental animals	Information included in the manuscript?	In which section is the information available? (Reagents and Tools Table, Materials and Methods, Figures, Data Availability Section)
**Laboratory animals or Model organisms:** Provide species, strain, sex, age, genetic modification status. Provide accession number in repository **OR** supplier name, catalog number, clone number, **OR** RRID.	Not Applicable	
**Animal observed in or captured from the field:** Provide species, sex, and age where possible**.**	Not Applicable	
Please detail **housing and husbandry conditions**.	Not Applicable	

Plants and microbes	Information included in the manuscript?	In which section is the information available? (Reagents and Tools Table, Materials and Methods, Figures, Data Availability Section)
**Plants:** provide species and strain, ecotype and cultivar where relevant, unique accession number if available, and source (including location for collected wild specimens).	Not Applicable	
**Microbes:** provide species and strain, unique accession number if available, and source.	Not Applicable	

Human research participants	Information included in the manuscript?	In which section is the information available? (Reagents and Tools Table, Materials and Methods, Figures, Data Availability Section)
If collected and within the bounds of privacy constraints report on age, sex and gender or ethnicity for all study participants.	Not Applicable	

Core facilities	Information included in the manuscript?	In which section is the information available? (Reagents and Tools Table, Materials and Methods, Figures, Data Availability Section)
If your work benefited from core facilities, was their service mentioned in the acknowledgments section?	Yes	Core Facility for Medical Proteomics, Tübingen

Design

Study protocol	Information included in the manuscript?	In which section is the information available? (Reagents and Tools Table, Materials and Methods, Figures, Data Availability Section)
If study protocol has been **pre-registered, provide DOI in the manuscript**. For clinical trials, provide the trial registration number **OR** cite DOI.	Not Applicable	
Report the **clinical trial registration number** (at ClinicalTrials.gov or equivalent), where applicable.	Not Applicable	

Laboratory protocol	Information included in the manuscript?	In which section is the information available? (Reagents and Tools Table, Materials and Methods, Figures, Data Availability Section)
Provide DOI OR other citation details if **external detailed step-by-step protocols** are available.	Yes	DOI: 10.5281/zenodo.17045153

Experimental study design and statistics	Information included in the manuscript?	In which section is the information available? (Reagents and Tools Table, Materials and Methods, Figures, Data Availability Section)
Include a statement about **sample size** estimate even if no statistical methods were used.	Yes	Sample sizes are mentioned for all data presented in the manuscript.
Were any steps taken to minimize the effects of subjective bias when allocating animals/samples to treatment (e.g. **randomization procedure**)? If yes, have they been described?	Not Applicable	
Include a statement about **blinding** even if no blinding was done.	Not Applicable	
Describe **inclusion/exclusion criteria** if samples or animals were excluded from the analysis. Were the criteria pre-established?<br><br>If sample or data points were omitted from analysis, report if this was due to attrition or intentional exclusion and provide justification.	Not Applicable	
For every figure, are **statistical tests** justified as appropriate? Do the data meet the assumptions of the tests (e.g., normal distribution)? Describe any methods used to assess it. Is there an estimate of variation within each group of data? Is the variance similar between the groups that are being statistically compared?	Yes	Statistical methods are described in the manuscript.

Sample definition and in-laboratory replication	Information included in the manuscript?	In which section is the information available? (Reagents and Tools Table, Materials and Methods, Figures, Data Availability Section)
In the figure legends: state number of times the experiment was **replicated** in laboratory.	Yes	
In the figure legends: define whether data describe **technical or biological replicates**.	Yes	

Ethics

Ethics	Information included in the manuscript?	In which section is the information available? (Reagents and Tools Table, Materials and Methods, Figures, Data Availability Section)
Studies involving **human participants**: State details of **authority granting ethics approval** (IRB or equivalent committee(s), provide reference number for approval.	Not Applicable	
Studies involving **human participants**: Include a statement confirming that **informed consent** was obtained from all subjects and that the experiments conformed to the principles set out in the WMA Declaration of Helsinki and the Department of Health and Human Services Belmont Report.	Not Applicable	
Studies involving **human participants:** For publication of **patient photos**, include a statement confirming that consent to publish was obtained.	Not Applicable	
Studies involving experimental **animals**: State details of **authority granting ethics approval** (IRB or equivalent committee(s), provide reference number for approval. Include a statement of compliance with ethical regulations.	Not Applicable	
Studies involving **specimen and field samples:** State if relevant **permits** obtained, provide details of authority approving study; if none were required, explain why.	Not Applicable	

Dual Use Research of Concern (DURC)	Information included in the manuscript?	In which section is the information available? (Reagents and Tools Table, Materials and Methods, Figures, Data Availability Section)
Could your study fall under dual use research restrictions? Please check biosecurity documents and list of select agents and toxins (CDC): https://www.selectagents.gov/sat/list.htm	Not Applicable	
If you used a select agent, is the security level of the lab appropriate and reported in the manuscript?	Not Applicable	
If a study is subject to dual use research of concern regulations, is the name of the **authority granting approval and reference number** for the regulatory approval provided in the manuscript?	Not Applicable	

Reporting

The MDAR framework recommends adoption of discipline-specific guidelines, established and endorsed through community initiatives. Journals have their own policy about requiring specific guidelines and recommendations to complement MDAR.

Adherence to community standards	Information included in the manuscript?	In which section is the information available? (Reagents and Tools Table, Materials and Methods, Figures, Data Availability Section)
State if relevant guidelines or checklists (e.g., **ICMJE, MIBBI, ARRIVE, PRISMA**) have been followed or provided.	Not Applicable	
For **tumor marker prognostic studies,** we recommend that you follow the **REMARK** reporting guidelines (see link list at top right). See author guidelines, under 'Reporting Guidelines'. Please confirm you have followed these guidelines.	Not Applicable	
For **phase II and III randomized controlled trials**, please refer to the **CONSORT** flow diagram (see link list at top right) and submit the CONSORT checklist (see link list at top right) with your submission. See author guidelines, under 'Reporting Guidelines'. Please confirm you have submitted this list.	Not Applicable	

Data Availability

Data availability	Information included in the manuscript?	In which section is the information available? (Reagents and Tools Table, Materials and Methods, Figures, Data Availability Section)
Have **primary datasets** been deposited according to the journal's guidelines (see 'Data Deposition' section) and the respective accession numbers provided in the Data Availability Section?	Yes	The mass spectrometry proteomics data have been deposited to the ProteomeXchange Consortium via the PRIDE with the dataset identifiers PXD048832, PXD048806 and PXD048808.
Were **human clinical and genomic datasets** deposited in a public access-controlled repository in accordance to ethical obligations to the patients and to the applicable consent agreement?	Not Applicable	
Are **computational models** that are central and integral to a study available without restrictions in a machine-readable form? Were the relevant accession numbers or links provided?	Yes	For structural modelling, the original AlphaFold2 training set was used as published by DeepMind. Additional code that we developed and used for the analysis is available at the following links: https://github.com/raimondilab/distogram_analysis ;
If publicly available data were reused, provide the respective **data citations in the reference list**.	Yes	Interactomic datasets from public databases were used (IntAct;Orchard et al., 2014)
